# Use of Floseal^®^, a human gelatine-thrombin matrix sealant, in surgery: a systematic review

**DOI:** 10.1186/1471-2482-14-111

**Published:** 2014-12-20

**Authors:** María Echave, Itziar Oyagüez, Miguel Angel Casado

**Affiliations:** Pharmacoeconomics & Outcomes Research Iberia, Pozuelo de Alarcón, 28224 Madrid, Spain

**Keywords:** Gelatin-thrombin-matrix sealant, Surgery, Floseal, Systematic review, Outcome

## Abstract

**Background:**

Surgical bleeding can be associated with an increased risk of morbidity and mortality across all surgical areas. Thus, numerous products have been developed to achieve haemostasis. A flowable haemostatic matrix such as Floseal^®^ can quickly and reliably stop bleeding across the full spectrum of bleeding scenarios. The aim of this study was to systematically review clinical and economic evidence regarding the use of Floseal^®^ in surgical procedures.

**Methods:**

An extensive literature search was conducted in PubMed, EMBASE, and the Cochrane Library over the period spanning 2003–2013 to identify publications related to Floseal^®^ use in all types of surgical procedures. Case reports and case series studies were excluded.

**Results:**

A total of 27 papers met the selection criteria and were analysed. In the studies, blood loss and the time to achieve haemostasis were the most reported outcomes used to assess the efficacy of Floseal^®^. The majority of published studies (64%) examined the use of Floseal^®^ compared with conventional methods (such as electrocautery or suturing). The remaining 36% of the studies evaluated the use of Floseal^®^ compared with other haemostatic agents, such as Surgicel^®^, Gelfoam^®^, and Hemostase^®^. FloSeal^®^ has been demonstrated to be an efficacious method in surgical procedures to reduce the time to achieve haemostasis, the frequency of intra- and postoperative bleeding, and the length of hospital stay, among other primary outcomes, resulting in less consumption of health resources.

**Conclusions:**

The majority of the selected studies confirmed that Floseal^®^ showed improvements over other haemostatic agents in achieving haemostasis and reducing blood loss.

**Electronic supplementary material:**

The online version of this article (doi:10.1186/1471-2482-14-111) contains supplementary material, which is available to authorized users.

## Background

Surgery procedures, independently of the type, usually follow a common approach. The major surgical steps are incision, dissection, exposure, resection, haemostasis, restoring anatomy and closure. Among the procedures, only anatomy differs, but the challenges are always the same. The typical surgical challenges are bleeding, healing complications, leakage and adhesion formation. Improperly addressing of these challenges, could impact on patient outcome such as haemorrhagic shock, blood replacement, longer hospital stay in case of bleeding.

Surgical bleeding, concretely, can be associated with an increased risk of morbidity and mortality across all surgical areas. In particular, bleeding complications arise in nearly 30% of surgeries [1].

Excessive bleeding complicates surgery and often leads to longer hospital stays, increased healthcare service utilisation, and higher healthcare costs, among other negative consequences [1].

The length of hospital stay is approximately 2–2.5 times longer for patients who require blood transfusion [2]. Efforts to control surgical bleeding and the use of blood transfusions are thus needed to reduce healthcare consumption and costs.

The most frequent methods typically used to achieve haemostasis were pressure (dressings) and sutures, but also numerous products have been developed to achieve the same aim by different ways, such as topical haemostatic agents (HA) (e.g., sponges), thrombin, gelatine-thrombin, fibrin glue, and other types of surgical sealants [3].

Although there is no consensus on how to best approach haemostasis, the number of options available to the surgeon continues to grow. Several factors are important when evaluating the quality of HAs and devices, but the most important are the ability of a product to achieve and maintain haemostasis and the speed with which bleeding is controlled [4].

Conventional methods for control bleeding are for example electrocautery, suturing, manual compression or ligatures, among others. Additionally, a broad variety of haemostatic agents such as vegetal-origin (Surgicel^®^, Tabotamp^®^, Hemostase^®^), fibrin sealants such as Tachosil^®^ or Tisseel^®^, different sponge products such as only composed of gelatine (Gelfoam^®^), or more specific techniques like polyvinyl alcohol sponge (Merocel^®^) and Infrared-sapphire coagulation which consist on light is converted into thermal energy thus causing coagulation and haemostasis, have been developed in the last decades.

Gelatine-thrombin matrix sealants are commonly used intra-operatively acting at the end stage of the coagulation cascade to facilitate fibrin formation, promoting coagulation and minimising blood loss. These agents are a mixture of a flowable gelatine matrix (bovine or porcine) and a human-derived thrombin component. For example, Floseal^®^ (Baxter Healthcare Corporation Fremont, CA 94555, USA) and Surgiflo^®^ (Ethicon Endo-Surgery, part of Johnson & Johnson Company, New Jersey 08876, USA) are composed of a bovine gelatine matrix and a porcine gelatine matrix respectively, and are typically prepared immediately before use and directly injected into the site of bleeding.

Floseal^®^ is indicated in surgical procedures as an adjunct to haemostasis when control of bleeding, ranging from oozing to spurting, by ligature or conventional procedures is ineffective or impractical.

The aim of the present study was to systematically review the clinical and economic literature regarding Floseal^®^ use in any type of surgical procedure.

## Methods

### Searching

PRISMA recommendations were followed using PRISMA checklist recorded on Additional file 1. An extensive systematic literature search was performed in MEDLINE using PubMed, in EMBASE using OVID, and in the Cochrane Library. English-language articles published during the last decade (from 1 January 2003 to 31 August 2013) were identified. The search targeted published studies presenting any clinical and/or economic type of evaluation of Floseal^®^ use during surgical procedures and in which Floseal^®^ was compared with at least one alternative. A secondary search among the citations of the articles retrieved in the initial search was performed to ensure that all relevant studies were included.

### Selection

The titles and abstracts of the studies identified by the search strategy were assessed for potential eligibility and were subsequently retained if they met the following inclusion criteria: (a) reporting clinical and/or economic outcomes, (b) describing any surgical approach, (c) including treatment with Floseal^®^ in comparison with conventional methods or with other HAs, and (d) written in either English or Spanish.

Abstract and poster publications were only considered if they were published within the past two years, as information about ongoing studies may be available as partially published research, such as conference abstracts [5]. In contrast, case series and case reports were excluded from the review.

### Interventions

Studies investigating surgical interventions using Floseal^®^ were included. Both laparoscopic and open surgeries were considered, and no surgical procedures were excluded, including investigations of Floseal^®^ use for epistaxis. The studies were then separately assessed based on surgery type.

### Search strategy

Details of the searches performed in MEDLINE and EMBASE are shown in Tables 1 and 2, respectively. The Cochrane Library was explored by entering ‘Floseal’ in the title, abstract, or keyword field.

**Table 1:** **Search strategy for the PubMed database**

	TERM	SEARCH DETAILS (24 September 2013)	NUMBER OF ARTICLES
1	Surgery	((((surgical procedures, operative [MeSH Terms]) OR general surgery [MeSH Terms]) OR surgery [Subheading]) OR Thoracic surgery [MeSH terms]) OR colorectal surgery [MeSH Terms]	2,885,426
2	Thrombin	(((thrombin [MeSH Terms]) OR thrombin [All Fields]) OR factor viiia [MeSH Terms]) OR factor viiia [A-ll Fields]	43,985
3	Matrix	((matrix bands [All Fields]) OR Matrix Metalloproteinases, Membrane-Associated [MeSH]) OR Hemostatic Matrix	8,096
4	Gelatin	((((((gelatin [MeSH Terms]) OR gelatin [Text Word]) OR gelatin sponge, absorbable [MeSH Terms]) OR gelatin sponge, absorbable [All Fields]) OR surgical sponges [MeSH Terms]) OR (surgical [All Fields] AND sponges [All Fields])) OR surgical sponges [All Fields]	22,894
5	Final fibrin	Fibrin [MeSH] OR fibrinogen [MeSH]	45,657
6	Sealant	Sealant [All Fields]	3,328
7	Floseal	Floseal [All Fields]	155
8	Humans	Humans [MeSH Terms] NOT animals [MeSH Terms:noexp]	11,498,554
9	Language	(english [lang]) OR Spanish[lang]	18,978,494
10		Matrix and thrombin	780
11		Matrix and gelatin	654
12		Matrix and sealant	109
13	Product Matrix	#10 OR #11 OR #12	1,370
14		Thrombin and matrix	780
15		Thrombin and gelatin	353
16		Thrombin and sealant	332
17	Product Thrombin	#14 OR #15 OR #16	1,294
18	Product	#13 OR #17	1,884
19	Product w/o fibrin	#18 NOT final fibrin	1,372
20	Final Product	#19 OR Floseal	1,403
21	Final 24Sep2013	20 AND Surgery AND Humans AND Language	176
22	Final 24Sep2013 w/o CR	#21 NOT “case reports”[Publication Type]	140
23	Final with dates	#22 (“2003/01/01”[PDAT]: “2013/08/31”[PDAT])	109

**Table 2:** **Search strategy for the EMBASE database**

	SEARCH DETAILS (24 September 2013)	NUMBER OF ARTICLES
1	exp surgery/ or exp colorectal surgery/ or exp general surgery/ or exp thorax surgery/	3,293,277
2	su.fs.	1,732,981
3	1 or 2	3,766,104
4	exp thrombin/	35,438
5	Thrombin.mp.	61,009
6	Factor viiia.mp. or exp blood clotting factor 8a/	912
7	4 or 5 or 6	61,516
8	Matrix bands.mp.	42
9	exp matrix metalloproteinase/	17,584
10	Hemostatic matrix.mp.	51
11	8 or 9 or 10	17,677
12	Gelatin.mp. or gelatin sponge/ or exp gelatin/	27,702
13	Gelatin sponge.mp.	2,463
14	Surgical sponges.mp. or exp surgical sponge/	953
15	(Surgical and sponges).mp. [mp = title, abstract, subject headings, heading word, drug trade name, original title, device manufacturer, drug manufacturer, device trade name, keyword]	600
16	12 or 13 or 14 or 15	28,917
17	exp fibrin/	18,545
18	exp fibrinogen/	46,855
19	17 or 18	60,613
20	sealant.mp.	6,172
21	floseal.mp.	375
22	exp human/	14,968,402
23	animal/	1,888,844
24	22 not 23	14,485,903
25	7 and 11	216
26	11 and 16	1,096
27	11 and 20	15
28	25 or 26 or 27	1,290
29	7 and 16	465
30	7 and 20	493
31	25 or 29 or 30	1,044
32	28 or 31	2,118
33	32 not 19	1,745
34	21 or 33	2,010
35	3 and 24 and 34	569
36	Limit 35 to (english or spanish)	540
37	exp case report/	1,986,006
38	36 not 37	460
39	Limit 38 to yr = ”2003 - 2012”	385

### Data extraction

Two reviewers screened the references based on the defined inclusion criteria and extracted the data. The data were collected by one author (ME) and checked by a second author (IO), and all disagreements were resolved through discussion.

## Results

**Figure 1:**
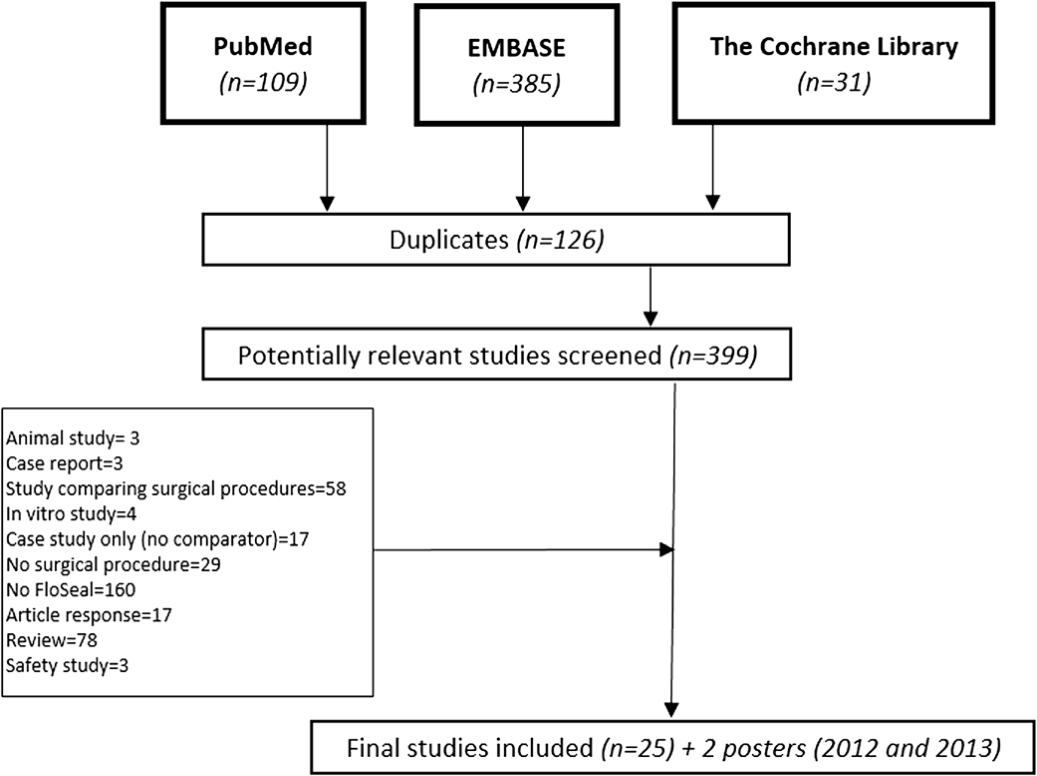
**Flow diagram of the selection process to identify studies to be included.**

A total of 525 potential publications from the last decade (2003–2013) were identified by the search (109 using MEDLINE, 385 using EMBASE, and 31 using the Cochrane Library). Among the 525 references, 126 were duplicates (24%) and were subsequently excluded. Additionally, 372 publications were excluded for the following main reasons: studies comparing surgical procedures or examining products other than Floseal^®^, case reports, and in vitro and animal studies. Figure 1 shows the flowchart of the selection process, indicating the potentially relevant studies identified, the studies retrieved for more detailed evaluation, the included studies, and the excluded studies [6–32].

### Included studies

**Figure 2:**
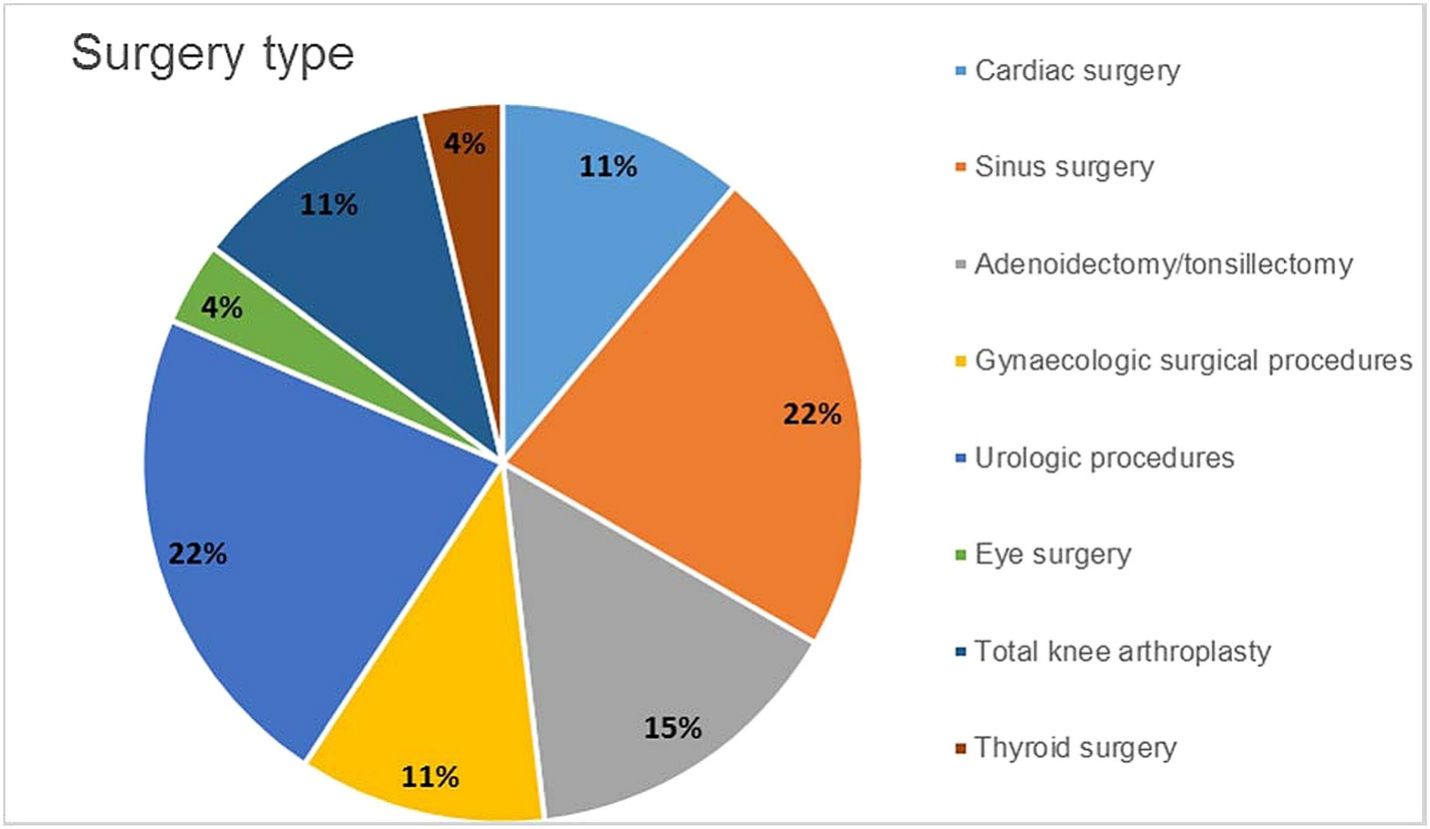
**Articles selected and included by surgery type.**

A total of 27 studies were ultimately identified and selected [6–33]. An overview of the characteristics of these 27 evaluations, including a total of 39,577 patients undergoing surgical procedures, is shown in Table 3. Six studies involved sinus surgery (22.2%); six, urologic procedures (22.2%); four, either adenoidectomy or tonsillectomy (14.9%); three, cardiac surgery (11.1%); three, gynaecologic surgical procedures (11.1%); and one, eye surgery (3.7%). Finally, three total knee arthroplasty (TKA) studies (11.1%) and one study on thyroid surgery (3.7%) were identified (Figure 2). Among the 27 studies included, 16 (59%) were randomized clinical trials.

**Table 3:** **Overview of the studies identified**

Surgery type	Author and year	Country	Study type (N)	Therapy
Cardiac surgery	Krishnan 2009 [6]	US	Retrospective chart study review (36,950)	Haemostatic agent
	Nasso 2009 [7]	Italy	Prospective randomised controlled trial (415)	Haemostatic agent
	Sugarman 2013 [8]	US	Economic evaluation	Haemostatic agent
Gyneacologic surgery	Angioli 2009 [23]	Italy	Prospective randomised controlled trial (20)	Conventional method
	Raga 2009 [24]	Spain	Prospective randomised controlled trial (50)	Conventional method
	Sönmezer 2013 [25]	Germany	Prospective randomised controlled trial (30)	Conventional method
Lacrimal surgery	Durrani 2007 [32]	UK	Cases and controls (20)	Conventional method
Orthopaedic surgery	Comadoll 2012 [11]	US	Retrospective chart study review (349)	Conventional method
	Kim 2012 [9]	US	Prospective randomised controlled trial (195)	Conventional method
	Velyvis 2012 [10]	US	Cases and controls (183)	Conventional method
Sinus Surgery	Baumann 2003 [19]	Switzerland	Cases and controls (100)	Haemostatic agent
	Beyea 2011 [21]	Canada	Prospective randomised controlled trial (20)	Haemostatic agent
	Chandra 2003 [16]	US	Prospective randomised controlled trial (20)	Haemostatic agent
	Chandra 2005 [17]	US	Prospective randomised controlled trial (18)	Haemostatic agent
	Jameson 2006 [20]	US	Prospective randomised controlled trial (45)	Conventional method
	Shrime 2007 [18]	US	Retrospective chart study review (172)	Conventional method
Thyroid surgery	Testini 2009 [22]	Italy	Prospective randomised controlled trial (155)	Haemostatic agent
Tonsillectomy/adenoidectomy	Blackmore 2008 [12]	UK	Prospective randomised controlled trial (30)	Conventional method
	Jo 2007 [15]	US	Prospective randomised controlled trial (68)	Conventional method
	Mathiasen 2004 [14]	US	Prospective randomised controlled trial (70)	Conventional method
	Mozet 2012 [13]	Germany	Prospective randomised controlled trial (176)	Conventional method
Urologic procedures	Gill 2005 [26]	US	Cases and controls (131)	Conventional method
	Guzzo 2009 [28]	US	Cases and controls (40)	Haemostatic agent
	Koni 2012 [27]	Turkey	Prospective randomised controlled trial (43)	Haemostatic agent
	Nogueira 2008 [29]	US	Cases and controls (35)	Haemostatic agent
	Pace 2010 [30]	Italy	Prospective randomised controlled trial (30)	Conventional method
	Waldert 2011 [31]	Austria	Cases and controls (142)	Conventional method

US: United States.

Fourteen studies (51.85%) were conducted in the U.S., and the remaining studies were from different countries, including Italy (4), the United Kingdom (2), Germany (2), Austria (1), Spain (1), Switzerland (1), Turkey (1), and Canada (1). In these studies, blood loss and the time to achieve haemostasis were the most reported outcomes used to assess treatment efficacy. Other outcomes included the length of hospital stay (LOS) and postoperative pain.

A synthesis of the selected studies is presented in Table 3. The population targeted in our review was any patient undergoing surgical procedures. To aggregate the results, studies included in this systematic review are grouped by surgical procedure.

**Table 4:** **Cardiac and vascular surgeries**

Author and year	Comparator and patients per arm (n)	Primary endpoint	Principal results
Krishnan 2009 [6]	Floseal^®^ (1,603)	Length of hospital stay	Floseal^®^ was associated with a lower likelihood of exceeding the expected LOS compared with baseline (OR = 0.791; p < 0.01)
	Surgicel^®^ + thrombin (17,507)		
	Gelfoam^®^ + thrombin (10,348)		
	Other (7,492)		
Nasso 2009 [9]	Floseal^®^ (209)	Rate of successful intraoperative haemostasis and time required for haemostasis	Significantly higher rates of successful haemostasis and a shorter time to haemostasis were observed in the Floseal^®^ group (p < 0.001 both)
	Topical haemostatic agent		
	(Surgicel^®^ or Gelfoam^®^) (206)		
Sugarman 2013 [8]	Economic comparison with 2009 Nasso study	Economic value when using Floseal^®^ to achieve haemostasis	The use of Floseal^®^ resulted in substantial net cost savings.

### Cardiac and vascular surgeries

Three studies identified in this review investigated the clinical and economic value of Floseal^®^ in cardiac and vascular procedures [6–8] (Table 4).

In a retrospective database analysis of a U.S. hospital database, Krishnan et al. [6] identified 36,950 cases of cardiovascular surgery with HA use between 2003 and 2006. Three treatment cohorts were compared against a baseline (n = 7,492) that consisted of the use of any single agent other than the following: Floseal^®^ (n = 1,603), Gelfoam^®^ + thrombin (n = 10,348), and Surgicel^®^ + thrombin (n = 17,507). Surgeries included in the analysis were open valvuloplasty, valve replacement, and coronary artery bypass. The primary outcome of the study was the LOS. The results showed that Floseal^®^ use was associated with a lower risk of exceeding the expected LOS (odds ratio = 0.791, p < 0.01) compared with baseline.

A prospective study conducted in Italy randomised 415 patients undergoing elective primary cardiac and/or thoracic aortic surgeries including Floseal^®^ use (n = 209) or an alternative topical HA (n = 206) chosen by the surgeon (Surgicel^®^ or Gelfoam^®^) [7]. Floseal^®^ was associated with a significantly higher rate of successful haemostasis and a shorter time to achieve haemostasis (p < 0.001 for both) in comparison with the other alternatives when conventional methods failed. Moreover, a significantly higher number of patients required blood transfusions in the treatment group (76%) compared with the Floseal^®^ group (56%) (p < 0.001). In addition, the number of blood units transfused was significantly higher in the treatment group than in the Floseal^®^ group (61 vs. 97 blood units, p < 0.001). Although the rates of revision for bleeding and of minor complications were not significantly different between groups in the overall cohort, there were significant differences in the subgroup of patients with evident intraoperative bleeding. In this subgroup of patients, 4.5% of the patients treated with Floseal^®^ required revision for bleeding, compared with 13.5% in the comparator group (p = 0.04). Also in this subgroup, minor postoperative complications, including renal failure, respiratory insufficiency, and inotropic support lasting for more than 24 hours, occurred in 20.9% of Floseal^®^-treated patients compared with 33.6% of patients in the comparator group (p = 0.04).

The third study was an abstract accepted for poster publication at the International Society For Pharmacoeconomics and Outcomes Research in May 2013 [8] that assessed the economic value of Floseal^®^ in the US based on the study endpoints and results of the Nasso study [7] and quantified the monetary costs associated with the efficient control of intraoperative bleeding and contributing complications. If FloSeal^®^ was the exclusive method used to achieve haemostasis in a hospital performing 600 mixed cardiac surgeries annually, outcomes would be improved and achieved cost savings due to the 242 hours saved in operating room and 33 major and 76 minor complications. Additionally, 54 surgical revisions for bleeding and 194 blood transfusions would be avoided. All of the clinical savings would result in total net annual savings ranging from $4.3-$7.4 million compared with costs in a comparator group (composed of 60.2% Surgicel^®^ and 39.8% Gelfoam^®^ treatments) in a US hospital setting.

### Orthopaedic surgery

Three publications described US studies performed in unilateral TKA comparing Floseal^®^ with standard of care such as the use of electrocautery, suturing, or manual compression (Table 5). All were US studies, two of them were prospective and the other one retrospective. In both the prospective studies the endpoint of measurement of blood loss through drain output was the same [9, 10], however the results were different.

**Table 5:** **Orthopaedic surgery**

Author and year	Comparator and patients per arm (n)	Primary endpoint	Principal results
Comadoll 2012 [11]	Floseal^®^ (184)	Pre- and postoperative decreases in haemoglobin decreases	Floseal^®^ resulted in less reduction in haemoglobin than did the use of conventional haemostatic methods (p < 0.0001).
	Conventional haemostatic methods (Electrocautery, suturing, or manual compression) (165)		
Kim 2012 [9]	Floseal^®^ (97)	Blood loss measured through drain output	Floseal^®^ had no demonstrable effect on blood loss, as measured through drain output.
	Electrocautery (99)		
Velyvis 2012 [10]	Floseal^®^ (83)	Blood loss measured through drain output	Floseal^®^ significantly reduced blood loss (p = 0.0001) and blood transfusion requirements (p = 0.07).
	Conventional method (100)		

One of the most recent studies [10] showed that the Floseal^®^ group (n = 83) had significantly less intraoperative blood loss (45.12 mL vs. 78.23 mL, p = 0.0001) and lower blood transfusion rates (p = 0.004) compared to standard of care (n = 100), whereas research performed by Kim et al. [9] did not find significant differences between Floseal^®^ (n = 97) and the control group (n = 99) (electrocautery followed by wound closure) in terms of drain output, transfusion rates, or postoperative pain.

In the retrospective study the principal endpoint was the measurement of haemoglobin levels, which is an indirect way to estimate blood loss in any surgery type [11]. The authors of this retrospective study concluded that decreases in haemoglobin, both pre- and post-surgery, were significantly reduced in the Floseal^®^ cohort (n = 184 vs n = 165 in control group) (the group difference in the maximal decrease in haemoglobin was 0.96 g/dL, p < 0.0001) [11].

### Tonsillectomy and adenoidectomy

Four prospective, randomised trials in tonsillectomy and adenoidectomy were identified and detailed on Table 6. Two of these trials were European studies that included all patients over 16 years of age [12, 13].

**Table 6:** **Tonsillectomy and adenoidectomy**

Author and year	Comparator and patients per arm (n)	Primary endpoint	Principal results
Jo 2007 [15]	Floseal^®^ (34)	Postoperative recovery time and morbidity	Floseal^®^ decreased postoperative pain and narcotic pain medication use (p < 0.05 both) and resulted in a faster return to regular diet and activity (p < 0.01 both). Also shorter operative times were observed (p < 0.0001) and less blotod loss (p < 0.05) with Floseal^®^
	Electrocautery (34)		
Blackmore 2008 [12]	Ligatures (one fossa randomised to Floseal^®^ and the other to ligatures) (30)	Postoperative pain	No reduction in pain in the Floseal^®^ group.
Mathiasen 2004 [14]	Floseal^®^ (35)	Time to haemostasis and blood loss	Floseal^®^ yielded significantly shorter times to haemostasis and less blood loss (p < 0.0001 both).
	Cautery (35)		
Mozet 2012 [13]	Floseal^®^ (89)	Handling, duration, and consumption of postoperative pain medication; wound healing; and rate of postoperative haemorrhage	The Floseal^®^ group had less postoperative pain (p = 0.074), a significantly shorter duration of pain medication use (p = 0.014), and reduced pain medication consumption/demand (p = 0.032). Not significant difference in postoperative haemorrhage.
	Bipolar electrocautery (87)		

The other two were US studies and included only children (mean age 7.1 years) undergoing adenoidectomy or adenotonsillectomy [14,15], respectively.

The two US studies included similar number of children (n = 70 and n = 68) and both concluded that Floseal^®^-treated patients experienced significantly less blood loss compared with EC-treated patients (2.5 vs. 29.4 mL, p < 0.001 [14]; 49.2 vs. 70.8 mL, p < 0.05 [15]).

In children undergoing adenoidectomy, the authors [14] also concluded that Floseal^®^ application (n = 35) yielded significantly shorter times to haemostasis (0.6 vs. 9.5 minutes, p < 0.001), significantly less subjective bleeding (0.0 vs. 2.0, as measured by visual analogue scale, p < 0.001), and operating surgeons experienced subjectively easier operations than electrocautery (n = 35) [14]. In addition to the endpoint of blood loss, one study performed from 2004–2005 concluded that adenotonsillectomy procedures in which Floseal^®^ was used had significantly shorter operating times compared with traditional electrocautery (16 vs. 31.2 minutes, p < 0.0001) [15]. In both US studies, Floseal^®^ application was also associated with significantly less pain (p < 0.05 for both) and an earlier return to a normal diet (p < 0.001 and p < 0.01) [14, 15].

The European studies were performed in patients over 16 years of age undergoing tonsillectomy. In Blackmore’s study, the patients (n = 30) were randomised to receive Floseal^®^ on one tonsil fossa, whereas ligatures were performed to achieve haemostasis in the other fossa, which acted as (the in-patient) control. The aim of this study was to evaluate postoperative pain with (n = 81) and without Floseal^®^ use (n = 89), although no statistically significant difference was found in postoperative pain scores when using Floseal^®^ [12]. The second European investigation randomised patients to receive Floseal^®^ or electrocautery after tonsillectomy [13]. The patients who received Floseal^®^ showed significantly improved wound healing throughout the postoperative period, a trend of less postoperative pain (not significant, NS), and a significantly shorter duration of pain medication use compared with electrocautery patients (9.5 vs. 11.6 days, p = 0.014), as well as reduced pain medication consumption/demand (p = 0.032) [13].

### Sinus surgery

Six clinical evaluations of endoscopic sinus surgery (ESS) were identified, with different study designs (randomisation of each fossa or patient to a different treatment) and comparators to evaluate the role of Floseal^®^ in this surgery type (Table 7). Five of these 6 studies were completed in North America, and the other one was performed in Europe.

**Table 7:** **Sinus surgery**

Author and year	Comparator and patients per arm (n)	Primary endpoint	Principal results
Chandra 2003 [16]	Floseal^®^ (20)	Effects on mucosal healing	Not significant differences in the extent of surgery or the need for additional nasal packing. The Floseal^®^ groups showed increased granulation tissue (p = 0.007) and adhesion formation (0.006).
	Thrombin-soaked gelatine foam (20)		
Chandra 2005 [17]	Floseal^®^ (10)	Long-term follow-up of previous Floseal^®^ study group (Chandra 2003)	Higher overall incidences of adhesions (p = 0.013) and adhesions requiring lysis (p = 0.046) in the Floseal^®^ group.
	Thrombin-soaked gelatine foam (8)		
Baumann 2003 [19]	Floseal^®^ (50)	Intra- and postoperative bleeding, cost of application, and length of hospital stay	Equal intraoperative haemostasis in both groups. A 36% shorter length of hospital stay in the Floseal^®^ group. High postoperative comfort in the Floseal^®^ group. The higher costs of Floseal^®^ application were largely compensated for by the lower hospitalisation costs.
	Merocel^®^ (50)		
Jameson 2006 [20]	Floseal^®^ (43)	Bleeding and healing	A shorter time to the cessation of bleeding in the Floseal^®^ group (p = 0.028). Less crusting in the Floseal^®^ group at 1 week and significantly less pain on Floseal^®^-treated patients (p = 0.027)
	Saline-soaked neuropatties (47)		
Shrime 2007 [18]	Floseal^®^ (37)	Incidence and outcomes of and risk factors for synechia formation	A higher incidence of synechia formation in the Floseal^®^ group. Similar intra- and postoperative complications.
	Conventional method (135)		
Beyea 2011 [21]	Floseal^®^ (10)	Nasal bleeding	NS difference in blood loss between groups (p = 0.93).
	Hemostase^®^ (8)		

Chandra et al. evaluated the postoperative and long-term effects of Floseal^®^ (n = 20) in comparison with other thrombin-soaked gelatine foams (n = 20) [16, 17]. The authors concluded that the Floseal^®^ group showed significantly increased granulation tissue (p = 0.007) and adhesion formation (p = 0.006), which are the most common complications after ESS.

Shrime [18] also analysed ESS with (n = 37) and without (n = 135) Floseal^®^ application. Patients were followed for 1.3 years after ESS, and the authors concluded that a significantly higher incidence of synechia formation was detected in the Floseal^®^ group, resulting in a higher rate of revision procedures (18.9% vs. 6.7%, p = 0.009).

When Floseal^®^ was compared with Merocel^®^ (synthetic haemostatic sponge with tamponade effect for nasal packing) in ESS, the length of hospitalisation was 36% shorter in the Floseal^®^ group (n = 50), and patient satisfaction was reported to be much higher in these patients. Additionally, the removal of Merocel^®^ (n = 50) caused pain, which was absent during Floseal^®^ use (no statistical data were provided for this research). The authors concluded that although the cost per application of Floseal^®^ was €198, compared with €19 for Merocel^®^, this difference was largely compensated for by lower hospitalisation costs in the Swiss population [19].

In a prospective, randomised, double-blinded controlled study of 45 patients undergoing bilateral ESS, each side was randomly assigned either Floseal^®^ followed by saline-soaked neurosurgical patties or a control treatment of saline-soaked neurosurgical patties alone [20]. In all, 20 patients received the same treatment on both sides, whereas 25 patients received a different treatment on each side. Floseal^®^ treatment resulted in significantly reduced bleeding in the immediate postoperative period. Moreover, the average duration of bleeding in the recovery room for Floseal^®^ compared with nasal packing was reported to be 16.4 minutes and 30.8 minutes, respectively (p = 0.028). In addition, patients reported less pain on the Floseal^®^ side (p = 0.027) in postoperative diaries. At a one-week follow-up, sinuses treated with Floseal^®^ exhibited less crusting than those of controls (2.4% vs. 18.6%, p = 0.015), although this difference resolved by month 1.

In the most recent of the ESS studies identified, eighteen patients who underwent ESS were randomised to receive either Floseal^®^ (n = 10) or Hemostase^®^ (n = 8), a purified plant polysaccharide [21]. The primary outcome measure of the study was total operative blood loss. The study concluded that there were no significant differences in intraoperative bleeding between the two groups. The groups were also comparable in bleeding grade and the number of nasal pledgets used.

### Thyroidectomy

One Italian thyroid surgery study identified in this review (Table 8), included 155 patients between January 2005 and December 2007, and were randomised to receive one of the following procedures: the surgical procedure alone (n = 49), Tabotamp Fibrillar^®^ (an oxidised regenerated cellulose patch) (n = 52), or Floseal^®^ (n = 54) [22]. The mean operating time was significantly reduced in the Floseal^®^ group (105 minutes) in comparison with the other two groups (133 minutes, p = 0.02, for the surgical procedure alone; 122 minutes, p = 0.0003, for Tabotamp^®^). Additionally, significantly earlier wound drain removal and shorter postoperative hospital stays occurred in the Floseal^®^ group (p = 0.006 vs. the surgical procedure alone; p = 0.008 vs. Tabotamp^®^).

**Table 8:** **Other surgery types**

Surgery type	Author and year	Comparator and patients per arm (n)	Primary endpoint	Principal results
**Gynaecologic surgery**	Angioli 2009 [23]	Floseal^®^ (8)	Control of minor bleeding	Not significant differences in the time to haemostasis, blood loss, or the operating time.
		Control (bipolar forceps or carbon-dioxide laser) (12)		
	Raga 2009 [24]	Floseal^®^ (25)	Haemostatic efficacy	Less intra- and postoperative blood loss (p = 0.001) and a lower rate of transfusions (0% in the Floseal^®^ group) (p < 0.001) for patients treated with Floseal^®^. A shorter length of hospital stay in the Floseal^®^ group (p = 0.005).
		Isotonic sodium chloride (25)		
	Sönmezer 2013 [25]	Floseal^®^ (13)	Ovarian reserve and damage	During the first postoperative month, ovarian damage was significantly lower in the Floseal^®^ group (p < 0.001). However, at the third month after surgery, NS differences were found.
		Bipolar electrosurgical coagulation (15)		
**Lacrimal surgery**	Durrani 2007	Floseal^®^ (10)	Postoperative bleeding and patient comfort	Nine patients in the Floseal^®^ group had no or minimal bleeding; this finding was statistically significant at all three measured time points (immediately (p = 0.047), at 12 h (p = 0.006), and at 24 h after surgery (p = 0.005)). The Floseal^®^ group also had less postoperative discomfort (p = 0.0001).
		Without Floseal^®^ (10)		
**Thyroid surgery**	Testini 2009 [22]	Floseal^®^ (54)	Operating time and wound drain removal	A significantly shorter operating time in the Floseal^®^ group than in the other groups (p < 0.05). More rapid wound drain removal and a shorter length of hospital stay in the Floseal^®^ group compared with the other groups (p < 0.05 both). Not significant difference in postoperative morbidity.
		Surgical haemostasis (49)		
		Tabotamp (52)		

### Gynaecologic surgery

Three studies, from Italy, Spain, and Turkey, included patients undergoing gynaecologic surgery, specifically myomectomy or laparoscopic excision of endometriomas, to evaluate the role of Floseal^®^ in intraoperative blood loss and the time to haemostasis [23–25] (Table 8).

The comparators were different between the studies; thus, the results also differed. When Floseal^®^ (n = 25) was compared with isotonic sodium chloride (n = 25) [24], Floseal^®^ was significantly better due to less intraoperative blood loss (25 mL vs. 250 mL, p = 0.001) and no patients requiring transfusion, in comparison with 20% of the control group (p < 0.001). However, when the comparator was a carbon-dioxide laser or bipolar forceps, Floseal^®^ yielded a shorter, but not statistically significant, time to haemostasis; less blood loss; and a lower decrease in postoperative haemoglobin [23]. In the third case [25], bipolar electrosurgical coagulation (n = 15) was compared with Floseal^®^ (n = 15) to evaluate the effect on ovarian reserve in patients undergoing laparoscopic endometrioma surgery. Acute ovarian damage was less common in the Floseal^®^ group during the first postoperative month, but the ovarian reserve was replenished in the bipolar electrosurgical coagulation group by the third month.

### Urologic procedures

Six evaluations of urologic procedures were identified. All were clinical evaluations, two of which also aimed to estimate the cost savings of each alternative. The primary endpoints in all of these studies were different and all details were collected on Table 9.

**Table 9:** **Urologic procedures**

Author and year	Comparator and patients per arm (n)	Primary endpoint	Principal results
Gill 2005 [26]	Floseal^®^ (63)	Reducing haemorrhagic complications	NS differences in the mean warm ischaemia time (p = 0.55), blood loss (p = 0.36), the operating time, or the length of hospital stay. Floseal^®^ had significantly fewer overall complications (p = 0.008).
	No Floseal^®^ (laparoscopic suturing) (68)		
Guzzo 2009 [27]	Floseal^®^ (19)	Operating and warm ischaemia times, blood loss, postoperative transfusion rate, length of hospital stay, and costs	Similar safety and efficacy for the two alternatives, and Gelfoam^®^ was less expensive than Floseal^®^.
	Gelfoam^®^ (21)		
Koni 2012 [28]	Floseal^®^ (11)	Differences in complications	The use of haemostatic agents significantly reduced postoperative complications. Among haemostatic agents, TachoSil^®^ provided the best benefits in terms of postoperative complications.
	Tachosil^®^ (25)		
	No use of haemostatic agents (7)		
Nogueira 2008 [29]	Floseal^®^ (25)	Haemostasis and blood loss	The ischaemia time (p = 0.148) and blood loss (p = 0.518) were comparable between the two groups.
	Surgiflo^®^ (10)		
Pace 2010 [30]	Floseal^®^ (15)	Efficacy in achieving haemostasis	Statistically higher rates of successful haemostasis and a shorter time to haemostasis were observed in the Floseal^®^ group (p < 0.001 both).
	Infrared-sapphire coagulator (ISC) (15)		
Waldert 2011 [31]	Floseal^®^ (32)	Efficacy and cost-effectiveness of Floseal^®^ in preventing lymphocele development after pelvic lymphadenectomy	Floseal^®^ may be effective in reducing the likelihood of lymphocele formation after pelvic lymphadenectomy. Data suggest that Floseal^®^ is cost effective because it reduces the need for diagnostic TC scans, laparoscopic fenestration, and subsequent prolonged hospitalisation.
	Without Floseal^®^ (110)		

In two of the studies, the primary objectives were to determine the differences in complications after laparoscopic partial nephrectomy (LPN) with Floseal^®^ application compared with conventional methods or Tachosil^®^ or no use of an HA [27]. In the first case, the Floseal^®^ group had significantly fewer overall complications (37% vs. 16%, p = 0.008) [26], and in the second case, the HA significantly reduced postoperative complications. More specifically, Tachosil^®^ provided a greater benefit compared with Floseal^®^ [27].

Two American studies compared Floseal^®^ with two different HAs, Surgiflo^®^ and Gelfoam^®^, and the authors did not find significant differences in terms of the time to ischaemia or blood loss [28, 29]. Additionally, Guzzo et al. [28] concluded that the potential cost saved per case using Gelfoam^®^ (n = 21) as a substitute for Floseal^®^ (n = 19) was up to $450 at their institution when performing LPN. A different surgical procedure was evaluated in a study by Pace et al. Thirty patients diagnosed with renal cell carcinoma who were going to undergo lumbar renal enucleoresection were randomised to receive Floseal^®^ (n = 15) or an infrared-sapphire coagulator (ISC) (n = 15) [30]. The authors observed significantly higher rates of successful haemostasis and a shorter time to haemostasis (8.1 vs. 12.9 minutes, p < 0.001) in the Floseal^®^ group. Moreover, the intra- and postoperative average blood loss was lower in the Floseal^®^ group (25 vs. 46 mL, p < 0.05). In addition, wound drain removal occurred earlier, particularly the day after surgery (p = 0.04), and the LOS was shorter in patients receiving Floseal^®^ (2.5 in the Floseal^®^ group, in comparison with 3.5 days in the ISC group, p < 0.05), and both findings were statistically significant.

Finally, a single-centre, matched comparison of lymphadenectomies in extraperitoneal radical prostatectomy with (n = 32) and without (n = 110) Floseal^®^ use was performed in Austria [31]. The results demonstrated that of the 32 Floseal^®^-treated patients, one (3.1%) developed symptomatic lymphocele, in comparison with 16 of 110 (14.5%) non- Floseal^®^ cases. Four of these patients had to be treated with percutaneous puncture, and six needed drainage and subsequent laparoscopic fenestration. In a cost analysis using the 2011 Euro, the mean cost per patient in the Floseal^®^ group was €327 compared with the non- Floseal^®^ cost per patient of €553, resulting in an average difference of €226 per patient. The authors concluded that Floseal^®^ is cost effective due to its ability to reduce the likelihood of lymphocele formation and the need for diagnostic CT scans, laparoscopic fenestration, and subsequent prolonged hospitalisation.

### Lacrimal surgery

A British prospective study [32] was performed with the aim of evaluating the role of Floseal^®^ in terms of postoperative bleeding and patient comfort in patients undergoing external dacryocystorhinostomy (DCR) (Table 8). In particular, Floseal^®^ was used during surgery in ten patients undergoing DCR. Ten additional consecutive patients underwent DCR without Floseal^®^. Nine patients in the Floseal^®^ group had no or minimal bleeding, and this difference was statistically significant at all three measured time points (immediately (p = 0.047), at 12 h (p = 0.006), and at 24 h after surgery (p = 0.05)). The Floseal^®^ group also had less postoperative discomfort (p = 0.0001).

## Discussion

To our knowledge, this is the first systematic review examining all clinical and economic studies on Floseal^®^ use in all surgery types.

In order to avoid exclusion of studies related to sealants consisting of gelatine and matrix components it was important not to restrict the search strategy too much. The large number of articles identified in our literature review could signify the relevance of research in the field of surgical sealants.

It is important to mention that Floseal^®^ has been evaluated in a wide variety of studies that had to be excluded from this review due to the inclusion criteria. Of the studies collected, nearly 40% of the studies were discarded. 58 studies were excluded because they included Floseal^®^ application in their surgical protocols but were designed to evaluate different surgical techniques, such as open or laparoscopic surgery but not to specifically evaluate the effectiveness of Floseal^®^.

Some limitations can be taken into account concerning this review. Some of them common to any other systematic reviews, were inherent to the methodology used. During the present review we faced the difficulty to determine all of the ways of describing Floseal^®^ (such as a glue or matrix, among others), and therefore, certain studies may have been missed despite our best efforts.

However, this risk should be minimal because of the large number of articles that were initially found.

Due to the scarce publications existing in specific surgical interventions such as lacrimal, thyroid, orthopedic and gynecological surgery, and the small patient sample size of them, any interpretation and/or extrapolation of the results should be precautionary done.

Additionally, a few studies that evaluated the efficacy of Floseal^®^ were not identified during the initial literature search due to restriction of the publication dates established in this research. However, during the secondary search among the citations of the articles retrieved in the primary search, it was observed that three studies were mentioned in many of the reviewed articles. These three important studies evaluated the role of Floseal^®^ in ESS, transsphenoidal pituitary surgery, and cardiac surgery [33–35] and concluded that Floseal^®^ use provided statistically significantly better results in terms of control of postoperative bleeding and the time to achieve haemostasis than did the comparators evaluated (cases study [33], standard of care [34] and Gelfoam^®^ [35]).

This review reflects the difficulty of comparing results because all 27 finally identified studies revealed a wide variety of surgery types (cardiac surgery, ESS, LPN, and adenoidectomy), and comparators (electrocautery, conventional methods, and other HAs). Additionally among same surgical procedure, also study designs (prospective, randomised clinical trials, cases and controls, retrospective chart reviews, and case studies) were varied and primary and secondary endpoints (the time to achieve haemostasis, blood loss, patient comfort after surgery, and pain control), so it implied and extra difficulty to compare results obtained in this research”.

Just only one study included in this review, evaluated the economic value of Floseal^®^ [8], concretely in cardiac surgery. Regarding thyroid surgery, the authors suggested that the costs associated with Floseal^®^ therapy are likely to be offset by a shorter postoperative stay and reduced time in the operating theatre [22]. The clinical benefits provided by Floseal^®^, in the majority of cases mentioned here, would probably yield to efficient healthcare resource use and lead to cost savings. However, this cannot be extrapolated if economic evaluations are not performed.

Additionally, fifteen studies evaluated the role of Floseal^®^ in different surgery types, but these studies were excluded because no comparator was included. The majority of these studies (53%) were evaluations of urologic procedures, and the authors concluded that Floseal^®^ rapidly controlled strong bleeding without suturing, which is highly advantageous to avoid the complicated technique required for suturing small bleeding vessels [36–41].

Significantly better results for Floseal^®^ were also identified in 18 of 26 studies (69.23%), and in the majority of the remaining studies, Floseal^®^ was found not to be inferior to the comparator in terms of effectiveness. An increasing number of studies have confirmed the effectiveness of Floseal^®^ in achieving haemostasis and reducing blood loss in patients undergoing surgical procedures. Reduction of both intra- and postoperative blood loss would reduce the need for blood transfusions and the LOS, and thus greatly reduce costs.

Fifteen of the 26 clinical evaluations considered in this review (with the exception of an economic study [8]) included 50 patients or more, and 12 of the studies had sample sizes of less than 50 patients. Considering the studies with <50 patients, 66.6% (8) of the results obtained were not significantly better for Floseal^®^, and the remaining 33.4% (4) were significantly better. However, 86.6% (13 of 15) of the studies with sample sizes of ≥50 patients obtained significantly better results for Floseal^®^ compared with alternative treatment. Another way to classify the selected studies is by study design: nearly 60% (16) of the 26 included clinical evaluations were prospective, randomised clinical trials, and 26% (7) were case–control studies. Additionally, 14% (3) of the clinical evaluations were retrospective chart reviews.

## Conclusion

Floseal^®^ has been demonstrated to be an efficacious alternative method in surgical procedures to obtain a complete and stable haemostasis and also in terms of reducing the time to achieve it, intra- and postoperative bleeding, and the length of hospital stay, among other primary outcomes, resulting in less consumption of health resources. Reduction in healthcare resource use seems to be associated with a decrease in patient management costs; thus, the use of Floseal^®^ might even yield cost savings.

In any case, economic evaluations could be interesting to assess the resource consumption associated to Floseal^®^ utilization. The development of further studies at local level should be performed to confirm that the costs of using Floseal^®^ would be balanced by a reduction in the use of health resources.

## Electronic supplementary material

Additional file 1: **PRISMA 2009 Checklist.** (DOCX 26 KB)
